# Fenbendazole induces pyroptosis in breast cancer cells through HK2/caspase-3/GSDME signaling pathway

**DOI:** 10.3389/fphar.2025.1596694

**Published:** 2025-07-18

**Authors:** Tingting Pan, Shengqi Jin, Xiaoxia Huang, Xin Xin, Qiming Xing, Wenhui Yang, Jing Dong, Lin Li

**Affiliations:** College of Animal Science and Veterinary Medicine, Shenyang Agricultural University, Shenyang, China

**Keywords:** breast cancer, fenbendazole (FBZ), pyroptosis, HK2, caspase-3, GSDME

## Abstract

**Introduction:**

Pyroptosis, a gasdermin (GSDM) - mediated programmed cell death associated with inflammation, has emerged as a promising strategy for cancer therapy. Metabolic reprogramming, a hallmark of cancer, presents potential targets for malignancy intervention. Fenbendazole (FBZ), a safe and inexpensive antiparasitic drug, has shown antitumor activities, but its underlying mechanisms remain unclear.

**Methods:**

We investigated the effects of FBZ on mouse mammary carcinoma cells in vitro using CCK - 8 assays, qPCR, Western blotting, and LDH release assays. Pyroptotic morphology was observed by microscopy. *In vivo*, we evaluated the antitumor efficacy of FBZ in a mouse mammary carcinoma model, analyzing tumor volume, weight, and histopathology. The involvement of the caspase - GSDM pathway and glycolysis (via hexokinase 2, HK2) was explored.

**Results:**

*In vitro*, FBZ dose - dependently inhibited cell viability, induced pyroptotic morphological changes (e.g., cell swelling and membrane pore formation), upregulated pyroptosis markers (cleaved caspase - 3, GSDME - NT, IL - 1β), and suppressed glycolysis by downregulating HK2. In vivo, FBZ treatment significantly reduced tumor volume and weight, with minimal systemic toxicity. Mechanistically, FBZ activated the caspase - 3/GSDME pathway and inhibited HK2 - dependent glycolysis.

**Conclusion:**

Our findings reveal that FBZ suppresses tumor growth by inducing pyroptosis and inhibiting glycolysis via HK2 downregulation. This study uncovers a novel mechanism for FBZ’s antitumor effects and highlights HK2 as a critical link between metabolism and cell death, suggesting FBZ as a potential candidate for cancer therapy.

## Graphical Abstract

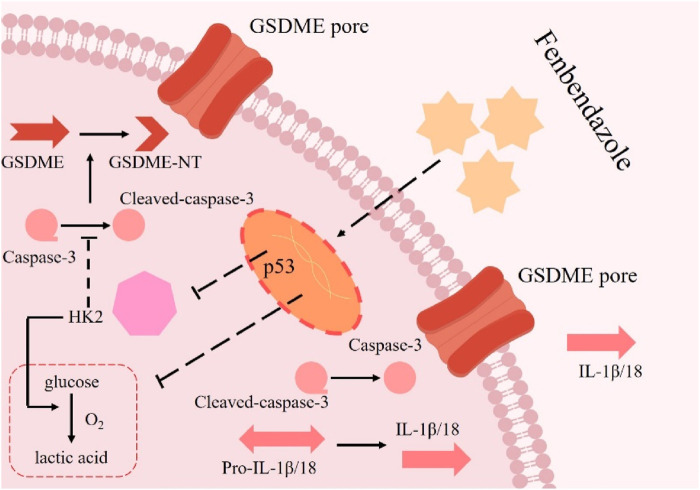

## 1 Introduction

Breast cancer stands as the most prevalent malignancy among women, posing a significant threat to female health (Harbeck and Gnant, 2017). Surgical resection remains the cornerstone of treatment; however, complete removal of malignant tumors often presents challenges (Waks and Winer, 2019). Neoadjuvant chemotherapy, administered before surgical tumor removal, has emerged as a strategic approach. Its objectives include converting initially inoperable tumors into resectable ones, facilitating conservative surgical options, and enhancing long-term patient outcomes (Gao et al., 2024). Nevertheless, the treatment landscape is complicated by the substantial toxicities associated with chemotherapeutic agents and the intricate mechanisms of drug resistance, frequently resulting in suboptimal therapeutic responses (Qu et al., 2022). Consequently, the identification of novel therapeutic targets and the development of innovative anticancer drugs are imperative for advancing breast cancer treatment.

Pyroptosis, a mode of regulated cell death, represents a highly coordinated pro-inflammatory response. Characterized by rapid cell swelling and lysis, it culminates in the release of pro-inflammatory cytokines (e.g., IL-1β, IL-18) and cellular contents into the extracellular space (Gao et al., 2022). Unlike apoptosis, this process is inherently inflammatory, with profound implications for tumor microenvironment modulation (Zapater et al., 2022). At the molecular level, gasdermin (GSDM) proteins serve as central effectors. Structurally, GSDMs consist of an N-terminal pore-forming domain and a self-inhibitory C-terminal domain linked by a flexible linker (Yu et al., 2021). Subtypes such as GSDMA, GSDMB, GSDMC, and GSDMD share sequence homology but exhibit context-dependent functions. All GSDMs operate under a conserved “auto-inhibition - cleavage activation” mechanism: upon detection of PAMPs or DAMPs, inflammasomes assemble to activate caspases (e.g., caspase-1, -4, -5, -11), which cleave GSDMs at their N-terminal linker regions. The released N-terminal fragments then oligomerize, insert into the cell membrane, and form pores that drive pyroptotic cell death (Rao et al., 2022). Notably, GSDME—a GSDM subtype predominantly expressed in epithelial cells—stands out for its reliance on caspase-3 for activation, a pathway implicated in cancer cell fate decisions (Rao et al., 2022). Emerging evidence highlights pyroptosis as a therapeutic target in oncology. For example, a study in glioblastoma demonstrated that benzimidazole ABZ induces pyroptosis via caspase-1-mediated GSDMD cleavage (Ren et al., 2022). However, whether FBZ—a structurally related benzimidazole—exerts anti-breast cancer effects through GSDM-dependent pyroptosis, particularly via the caspase-3/GSDME axis, remains undefined.

Glycolytic reprogramming represents a fundamental hallmark of cancer and plays a pivotal role in tumor progression (Ganapathy-Kanniappan and Geschwind, 2013). In cancer cells, dysregulated glucose metabolism not only supports self-proliferation, angiogenesis, lymphangiogenesis, and metastasis but also influences immune escape, prognosis assessment, and therapeutic responses (Ganapathy-Kanniappan and Geschwind, 2013). Mammals express four hexokinase (HK) isoforms (HK1, HK2, HK3, and HK4), among which HK2 is markedly upregulated in tumor tissues compared to normal counterparts (Zapater et al., 2022). Emerging evidence indicates that HK2 deficiency attenuates breast cancer metastasis (Zapater et al., 2022). Beyond its canonical role in glycolysis, HK2 exhibits non-metabolic functions. Although previous studies have implicated HK2 in regulating cell death, the underlying molecular mechanisms remain incompletely understood.

**FIGURE 1:**
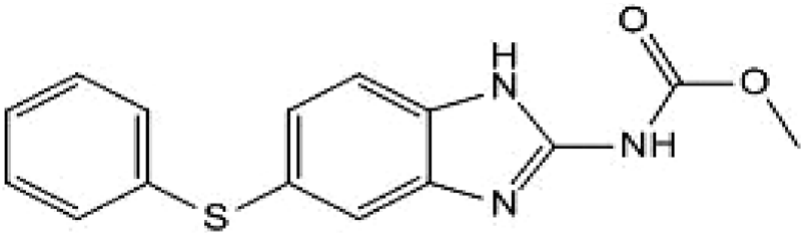
Chemical structure of FBZ.

Given the high failure rate, substantial costs, and lengthy timelines associated with *de novo* drug discovery, the repurposing of already approved drugs has gained significant traction in oncology research (Taneja et al., 2017). Fenbendazole (FBZ; Figure 1), a broad-spectrum anthelmintic agent approved for veterinary use across diverse animal species, has recently been shown to induce apoptosis and disrupt microtubule dynamics as mechanisms of its antitumor activity (Leach et al., 2024). However, whether FBZ can modulate pyroptosis to inhibit breast cancer progression remains undefined. Elucidating the detailed anticancer mechanisms of FBZ is therefore critical to optimizing its therapeutic potential for breast cancer treatment.

## 2 Results

### 2.1 FBZ inhibited the growth of mouse breast cancer xenograft by pyroptosis

**FIGURE 2:**
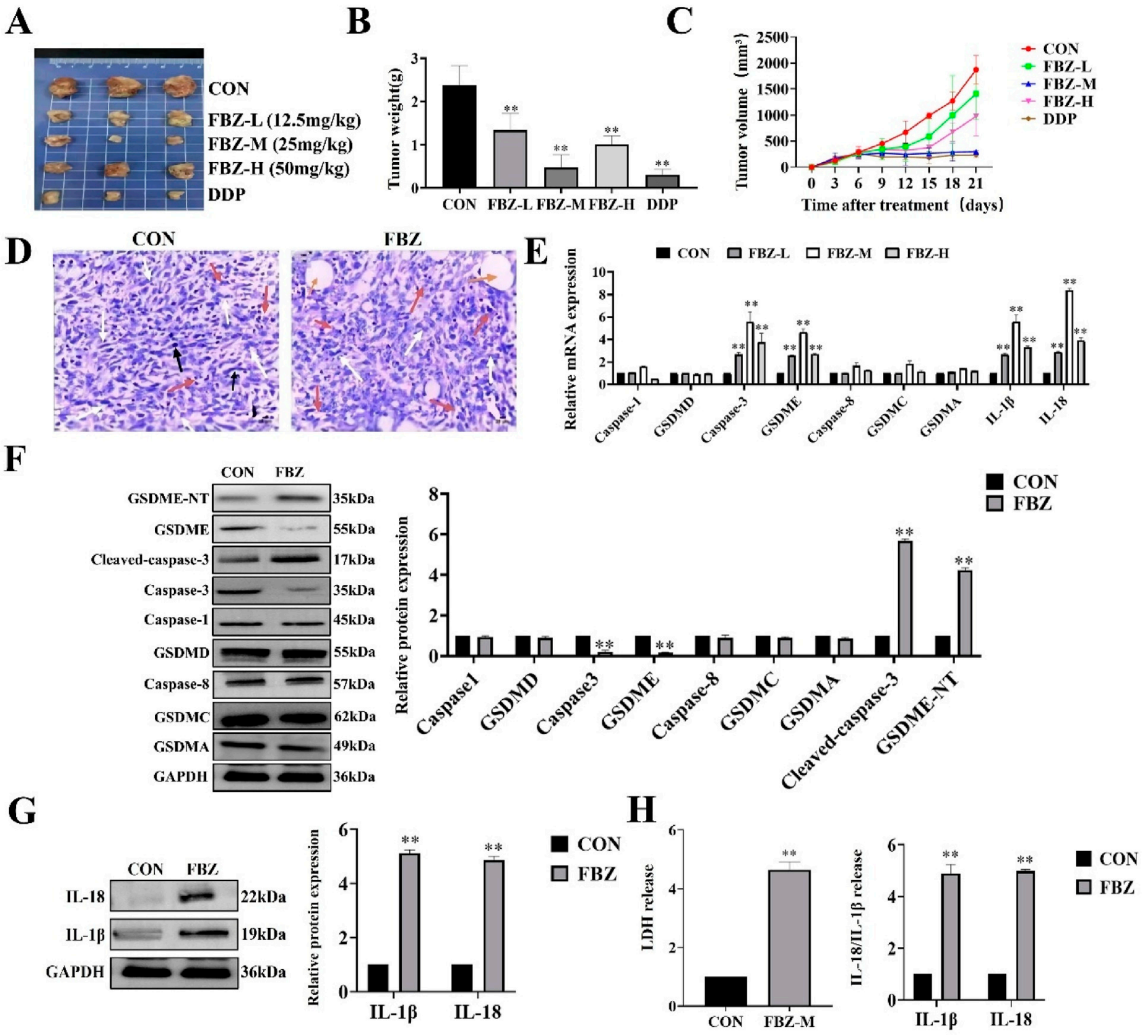
FBZ inhibits mouse mammary tumor growth via pyroptosis-related pathways. **(A)** Representative gross tumor images. Groups: CON (Control), FBZ-L (FBZ, 12.5 mg/kg), FBZ-M (FBZ, 25 mg/kg), FBZ-H (FBZ, 50 mg/kg), DDP (Cisplatin, positive control). **(B)** Tumor weight quantification. Data: Mean ± SD, n = 6 mice/group. Statistics: *P* < 0.01 vs. CON (one-way ANOVA). **(C)** Tumor volume kinetics. Measurement: Assessed every 3 days for 21 days. **(D)** H&E staining of tumor sections (×200 magnification, scale bar = 50 μm). pyroptotic shrinkage (white arrows), Necrotic foci (yellow circles), Mitotic figures (black arrows). **(E)** Pyroptosis-related mRNA expression (qPCR). Normalization: GAPDH (internal control), n = 3. **(F)** Western Blot analysis of pyroptosis proteins in tumor tissue. Detected proteins: GSDME-NT, Cleaved-caspase-3, Caspase-3, GSDMD, Caspase-8, GSDMC, GSDMA, GAPDH (loading control). Quantification: Relative protein expression normalized to GAPDH, n = 3. **(G)** WB analysis of IL-1β and IL-18 protein levels. Data: Mean ± SD, n = 3. **(H)** LDH activity and cytokine release in tumor supernatants. Sample processing: Tumors were rinsed with PBS, minced, homogenized on ice, and centrifuged (12,000 × g, 15 min, 4°C). Significance: P < 0.01 vs. CON.

To directly verify the effect of FBZ on breast cancer graft formation and growth in mice, mouse mammary carcinoma EMT6 cells were inoculated into Balb/c mice for tumorigenicity assessment. Female Balb/c tumor-bearing mice (6–8 weeks old) were randomized into six groups (n = 5 per group): control, normol, FBZ (12.5, 25, and 50 mg/kg), and cisplatin (positive control). Drugs were administered via intraperitoneal injection every 3 days for 21 days (7 total doses), with FBZ dissolved in DMSO and diluted in normal saline to the specified concentrations and cisplatin prepared at a clinical equivalent dose in normal saline. Tumor volume and mouse body weight were monitored as endpoints to balance efficacy evaluation and animal welfare. By weighing tumors and observing their macroscopic appearance, we found that all FBZ - treated groups exhibited tumor growth inhibition. Notably, the medium - dose FBZ group showed the most prominent inhibitory effect, which was comparable to that of cisplatin (DDP) (Figures 2A–C). In HE - stained sections, in the medium - dose FBZ group, compared with the CON group, it was observed that FBZ disrupted the structural integrity of tumor cells. Tumor boundaries became blurred, nuclei showed obvious lysis, and a large number of pyroptotic - like cells were visible (the specific cell characteristics pointed by arrows in the figure were further defined as manifestations related to such changes) (Figure 2D). We detected the relative mRNA expression related to pyroptosis using quantitative real - time PCR (qPCR) and analyzed protein expression via Western Blot (WB) (Figures 2E–G). The results demonstrated that FBZ treatment led to highly significant upregulation of the relative mRNA and protein expression levels of GSDME, caspase - 3, IL - 18, and IL - 1β (P < 0.01). Additionally, we investigated the impact of FBZ on the release of lactate dehydrogenase (LDH), IL - 18, and IL - 1β from mouse breast cancer xenografts (Figure 2H). The results showed that FBZ-M significantly promoted the release of LDH, IL-18 and IL-1β (*P* < 0.01). Therefore, we hypothesize that FBZ may induce pyroptosis through the caspase - 3/GSDME pathway, thereby exerting a suppressive effect on breast tumors.

### 2.2 Safety of FBZ in mice

**FIGURE 3:**
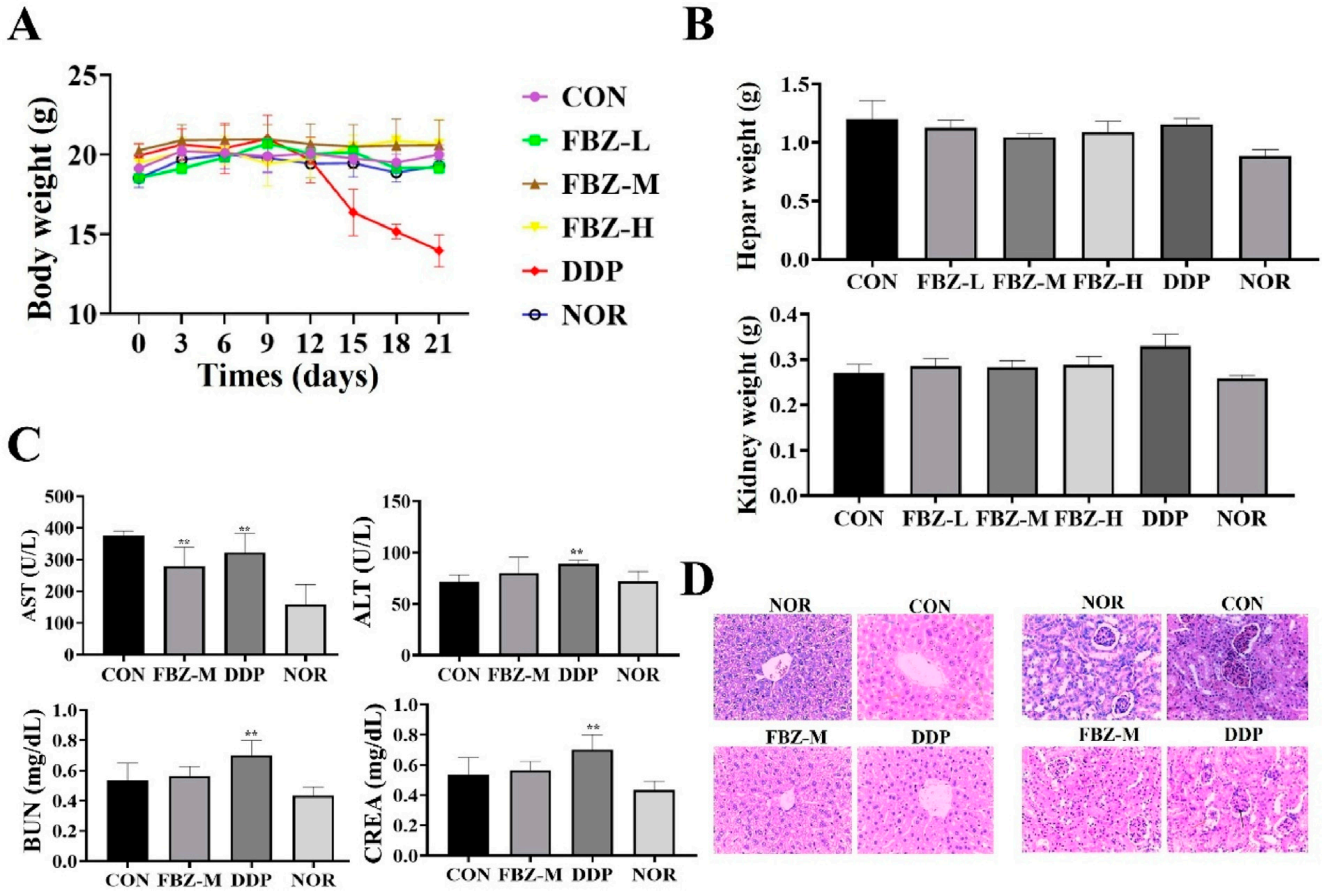
Safety assessment of fenbendazole (FBZ) in mouse mammary tumor model. **(A)** Longitudinal body weight monitoring. Groups: CON (Control), FBZ-L (FBZ, low dose), FBZ-M (FBZ, medium dose), FBZ-H (FBZ, high dose), DDP (Cisplatin, positive control), NOR (Normal, untreated). (n = 6, mean ± SD). **(B)** Liver and kidney weight quantification. Measurement: Absolute weights of liver (top) and kidney (bottom) at endpoint. Comparison: No significant change in FBZ groups vs. CON; DDP induced slight organ weight alterations (n = 6, mean ± SD). **(C)** Serum biochemical markers. Detected indices: AST (Aspartate Aminotransferase, U/L), ALT (Alanine Aminotransferase, U/L), BUN (Blood Urea Nitrogen, mg/dL), CREA (Creatinine, mg/dL). (n = 6, mean ± SD). **(D)** Histopathological analysis (H&E staining, ×200 magnification, scale bar = 50 μm). Tissues: Liver (top) and kidney (bottom) from FBZ-M, DDP, and NOR/CON groups. FBZ-M group: Normal liver lobule architecture and kidney glomerular/tubular structure. DDP group: Hepatocyte necrosis (liver, arrows), glomerular shrinkage, and tubular injury (kidney, arrows), confirming organ damage.

To assess FBZ safety, we monitored mouse body weights during administration (Figure 3A). Results showed no significant body weight differences between FBZ - treated and control groups. In contrast, mice in the DDP group started losing weight progressively from between day 9 and day 10 of administration and eventually succumbed to emaciation during treatment. Further evaluations of liver/kidney weights, histopathology, and liver/kidney function (Figures 3B–D) revealed FBZ exerted minimal - to - no impact on liver/kidney weights. Histologically, the liver had normal architecture with homogeneous staining; glomerular mesangial cells were intact and uniformly stained, and no abnormalities were detected in distal tubules, proximal tubules, or the interstitium. Serum alanine aminotransferase (ALT) levels in FBZ - treated mice were significantly lower than the control group (*P* < 0.01), while aspartate aminotransferase (AST), blood urea nitrogen (BUN), and creatinine (CREA) levels showed no marked differences vs. normal mice. Conversely, in the DDP group, liver/kidney weights were extremely significantly increased (*P* < 0.01). Hepatocyte nuclei displayed lesions like condensation, fragmentation, and lysis; glomerular volume enlarged, and the glomerular basement membrane thickened. Moreover, ALT, AST, BUN, and CREA concentrations were highly significantly elevated (*P* < 0.01). Collectively, these findings indicate DDP induces significant toxic - side effects, whereas FBZ demonstrates a favorable safety profile.

### 2.3 FBZ induced EMT6 cells pyroptosis

**FIGURE 4:**
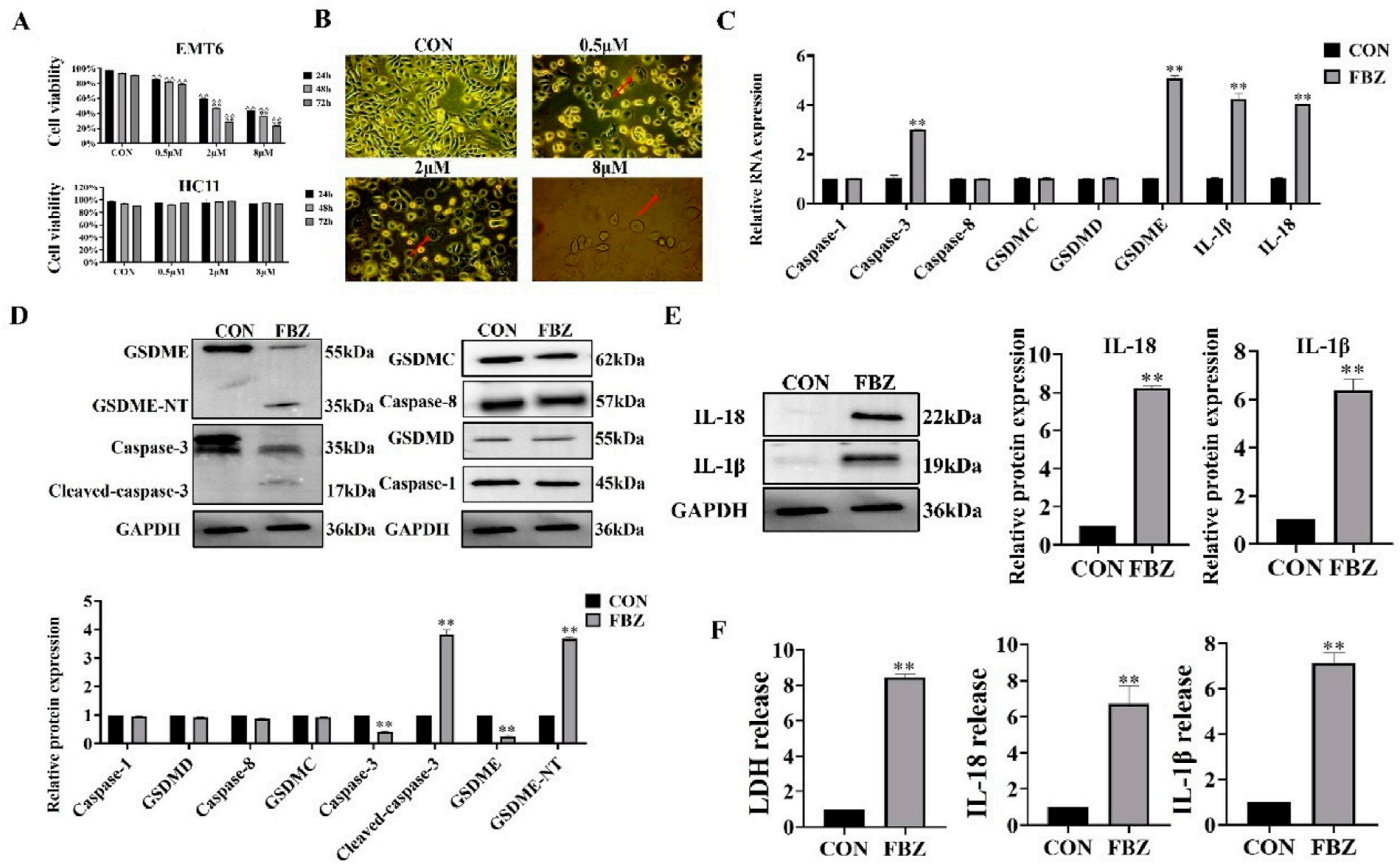
Fenbendazole (FBZ) induces pyroptosis in EMT6 breast cancer cells. **(A)** CCK-8 assay for cell proliferation. Cell lines: EMT6 (top) and HCT116 (bottom, negative control for FBZ sensitivity). Treatment: FBZ at 0.5–8 μM for 24 h (n = 6, mean ± SD). **(B)** Pyroptotic morphology observation (×200 magnification, scale bar = 50 μm). Phenotypes: CON (Control): Normal cell morphology. FBZ (2 μM, 24 h): Swelling, blistering (red arrows), characteristic of pyroptosis. **(C)** qPCR analysis of pyroptosis-related genes. Treatment: FBZ (2 μM, 24 h). (n = 3, mean ± SD, normalized to GAPDH). **(D)** Western Blot validation of pyroptosis execution. Treatment: FBZ (2 μM, 24 h) (n = 3, mean ± SD, normalized to GAPDH). **(E)** WB analysis of mature IL-1β/IL-18 secretion. Markers: IL-18 (22 kDa, mature), IL-1β (19 kDa, mature). Treatment: FBZ (2 μM, 24 h). (n = 3, mean ± SD, normalized to GAPDH). **(F)** LDH and cytokine release assays. Indices: LDH (cytotoxicity), IL-18, IL-1β (pyroptotic markers). Treatment: FBZ (2 μM, 24 h). (n = 3, mean ± SD).

In in vivo experiments, we found that FBZ inhibited mammary cancer xenografting in mice by pyroptosis and has a high safety profile. Next, we investigated whether FBZ could induce pyroptosis *in vitro* using the EMT6 cell line. The CCK - 8 assay revealed FBZ inhibited EMT6 cell proliferation in a time - and dose - dependent manner (*P* < 0.01) yet showed negligible toxicity to HC11 cells (Figure 4A), indicating FBZ can kill mammary tumor cells while maintaining safety for normal mouse mammary epithelial cells. Morphological observation found FBZ - treated EMT6 cells exhibited swelling, rupture, and large blister formation (Figure 4B), typical pyroptosis hallmarks. Assessing pyroptosis - related factors, FBZ significantly activated caspase - 3 and GSDME (P < 0.01), while changes in GSDMD, GSDMC, caspase - 1, and caspase - 8 were not statistically prominent, consistent with *in vivo* results (Figures 4C,D). Moreover, FBZ significantly enhanced lactate dehydrogenase (LDH), IL - 18, and IL - 1β release (Figures 4E,F), confirming functional pyroptotic pathway activation. Collectively, these data suggest a potential role for FBZ in inducing pyroptosis - like death in EMT6 cells, potentially mediated by caspase - 3/GSDME, while sparing HC11 mammary epithelial cells. This preliminary evidence highlights FBZ’s tumor - selective effects, warranting further investigation to definitively elucidate the underlying mechanisms.

### 2.4 FBZ-induced pyroptosis of EMT6 cells is dependent on GSDME

**FIGURE 5:**
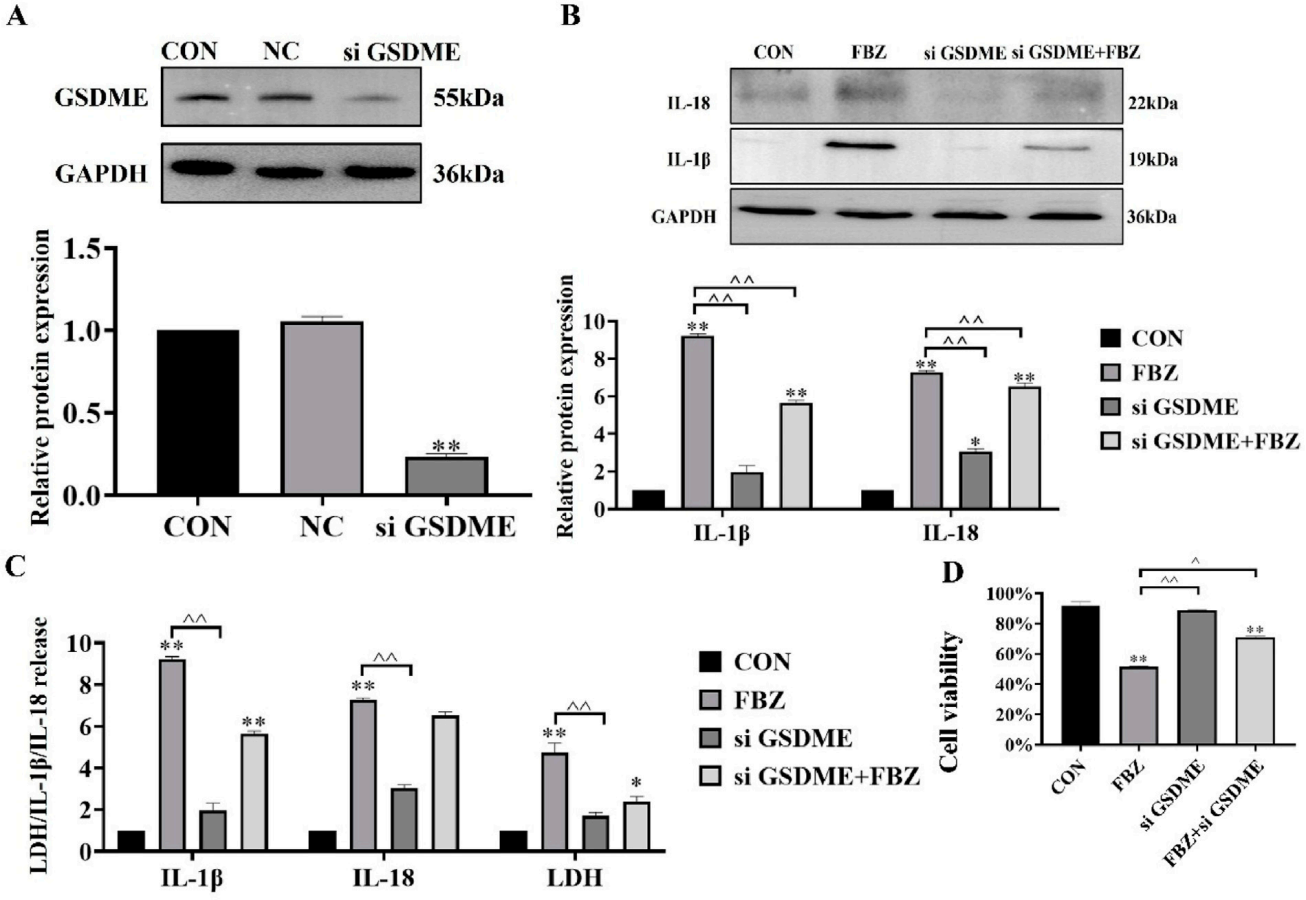
GSDME mediates FBZ-induced pyroptosis in EMT6 cells. **(A)** GSDME knockdown efficiency (Western Blot). Groups: CON (Control), NC (Negative Control), siGSDME (GSDME-targeting siRNA). Markers: GSDME (55 kDa), GAPDH (36 kDa, internal control). (P < 0.01 vs. CON/NC, n = 3, normalized to GAPDH). **(B)** IL-1β/IL-18 activation (2 μM FBZ treatment). Groups: CON, FBZ, siGSDME, siGSDME + FBZ. Markers: IL-18 (22 kDa, mature), IL-1β (19 kDa, mature), GAPDH (36 kDa). (P < 0.01 vs. FBZ, n = 3, normalized to GAPDH). **(C)** LDH/IL-1β/IL-18 release assays. Indices: LDH (cytotoxicity), IL-1β, IL-18 (pyroptotic markers). Treatment: 2 μM FBZ. (P < 0.01, n = 3). **(D)** Cell viability (CCK-8 assay). Groups: CON, FBZ, siGSDME, siGSDME + FBZ. (P < 0.01 vs. CON/FBZ, n = 6).

Building on prior findings that FBZ induces pyroptosis in EMT6 cells—with preferential cleavage of GSDME within the gasdermin family—we hypothesized GSDME acts as a key trigger for FBZ-mediated pyroptosis. To test this, we first knocked down GSDME in EMT6 cells using small interfering RNA (siRNA), achieving significant suppression of GSDME protein expression (*P* < 0.01; Figure 5A). Functional assays revealed that GSDME knockdown diminished FBZ-induced release of lactate dehydrogenase (LDH), mature IL-18, and IL-1β (*P* < 0.01; Figures 5B,C). Notably, GSDME silencing failed to reduce overall EMT6 cell death induced by FBZ (Figure 5D). We speculate that in the absence of GSDME-dependent pyroptosis, FBZ may instead trigger apoptosis as a compensatory cell death pathway, highlighting potential plasticity in FBZ’s mechanism of action.

### 2.5 The BAX/caspase-3 cascade was required for FBZ-induced GSDME pyroptosis

**FIGURE 6:**
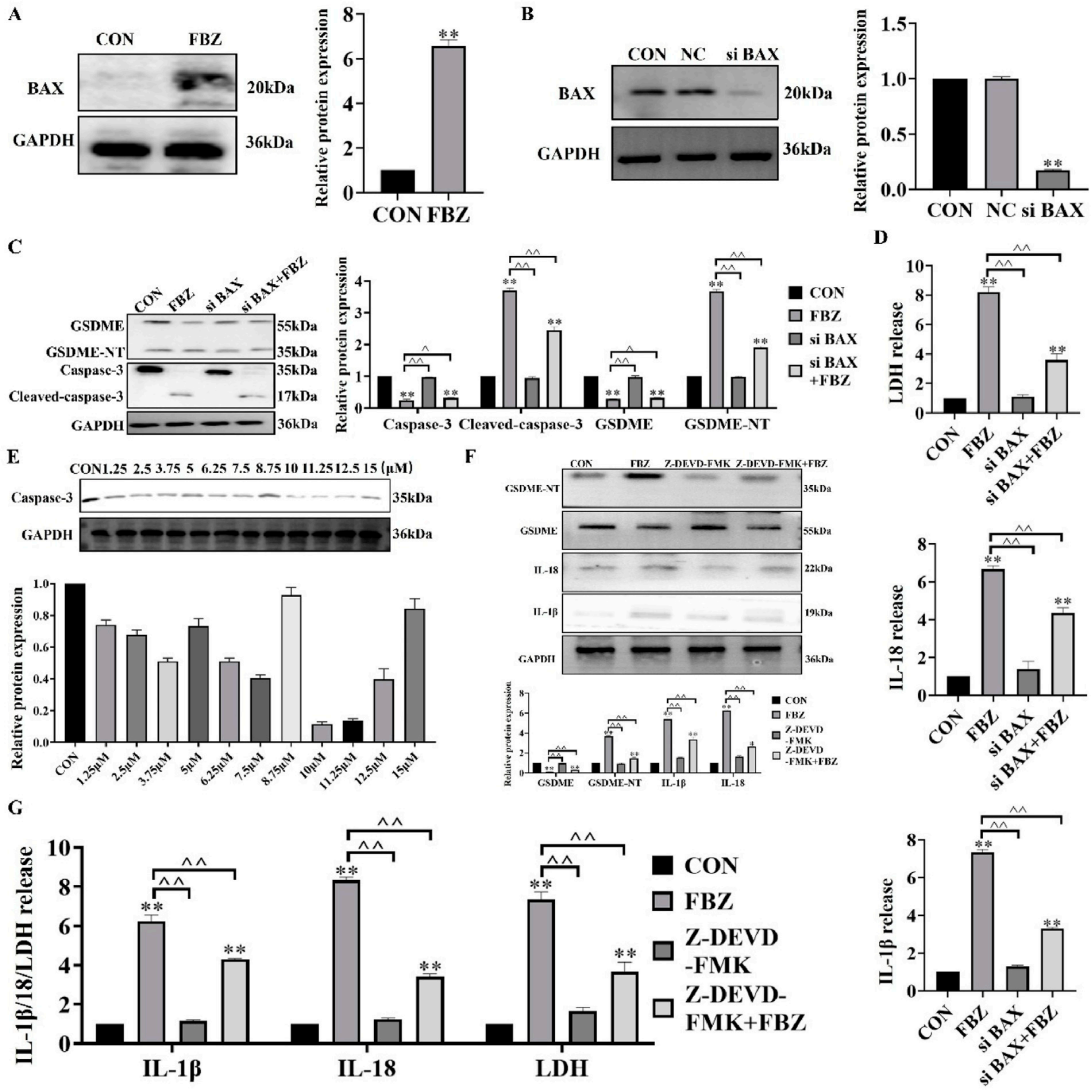
FBZ triggers GSDME-mediated pyroptosis via the BAX-caspase-3 cascade in EMT6 cells. **(A)** BAX protein upregulation (2 μM FBZ, 24 h). WB markers: BAX (20 kDa), GAPDH (36 kDa, internal control). (P < 0.05 vs. CON, n = 3, normalized to GAPDH). **(B)** BAX knockdown efficiency (siRNA). Groups: CON, NC (Negative Control), siBAX. WB markers: BAX (20 kDa), GAPDH (36 kDa). (*P* < 0.01 vs. CON/NC, n = 3, normalized to GAPDH). **(C)** Caspase-3/GSDME activation after BAX silencing (2 μM FBZ, 24 h). WB targets: GSDME (55 kDa), Cleaved-caspase-3 (17 kDa), GSDME-NT (35 kDa). (n = 3, normalized to GAPDH). **(D)** LDH release assay (2 μM FBZ, 24 h). Groups: CON, FBZ, siBAX, siBAX + FBZ. (P < 0.01 vs. FBZ, n = 3). **(E)** Screening of Z - DEVD - FMK concentrations. WB markers: Caspase-3 (35 kDa). (n = 3, normalized to GAPDH). **(F)** GSDME-NT detection after Z-DEVD-FMK + FBZ treatment. WB markers: GSDME-NT (35 kDa), GAPDH (36 kDa). (n = 3, normalized to GAPDH). **(G)** LDH/IL-1β/IL-18 release with caspase-3 inhibition (2 μM FBZ, 24 h). Groups: CON, FBZ, Z-DEVD-FMK, Z-FMK (inactive control), Z-DEVD-FMK + FBZ. (P < 0.01 vs. FBZ, n = 3).

Next, we examined the regulators upstream of GSDME. The WB results showed that FBZ significantly regulated the expression of bax in EMT6 cells (Figure 6A). The above study confirmed that FBZ could induce the activation of caspase-3 in EMT6 cells (Figure 4D). Based on these results and previous studies, we speculated that the Bax/caspase-3 cascade activated GSDME-induced pyroptosis. We knocked down bax expression in EMT6 cells with si RNA (Figure 6B). After degradation of bax, FBZ-induced activation of GSDME and caspase-3 was inhibited (Figure 6C). In addition, the release of LDH, IL-18 and IL-1β was inhibited (Figure 6D). We verified whether GSDME activation was via caspase-3 by using a caspase-3-specific inhibitor (Z-DEVD-FMK) (Figure 6E). The results showed that the protein expression of GSDME-NT and IL-18 and IL-1β was reduced by Z-DEVE-FMK treatment (Figure 6F), and the release of LDH, IL-18 and IL-1β was also reduced (Figure 6G). Collectively, these data demonstrate FBZ triggers GSDME cleavage through a BAX - caspase - 3 cascade, establishing a linear pathway for pyroptosis induction in EMT6 cells.

### 2.6 FBZ inhibited the p53/HK2 axis and aerobic glycolysis in EMT6 cells

**FIGURE 7:**
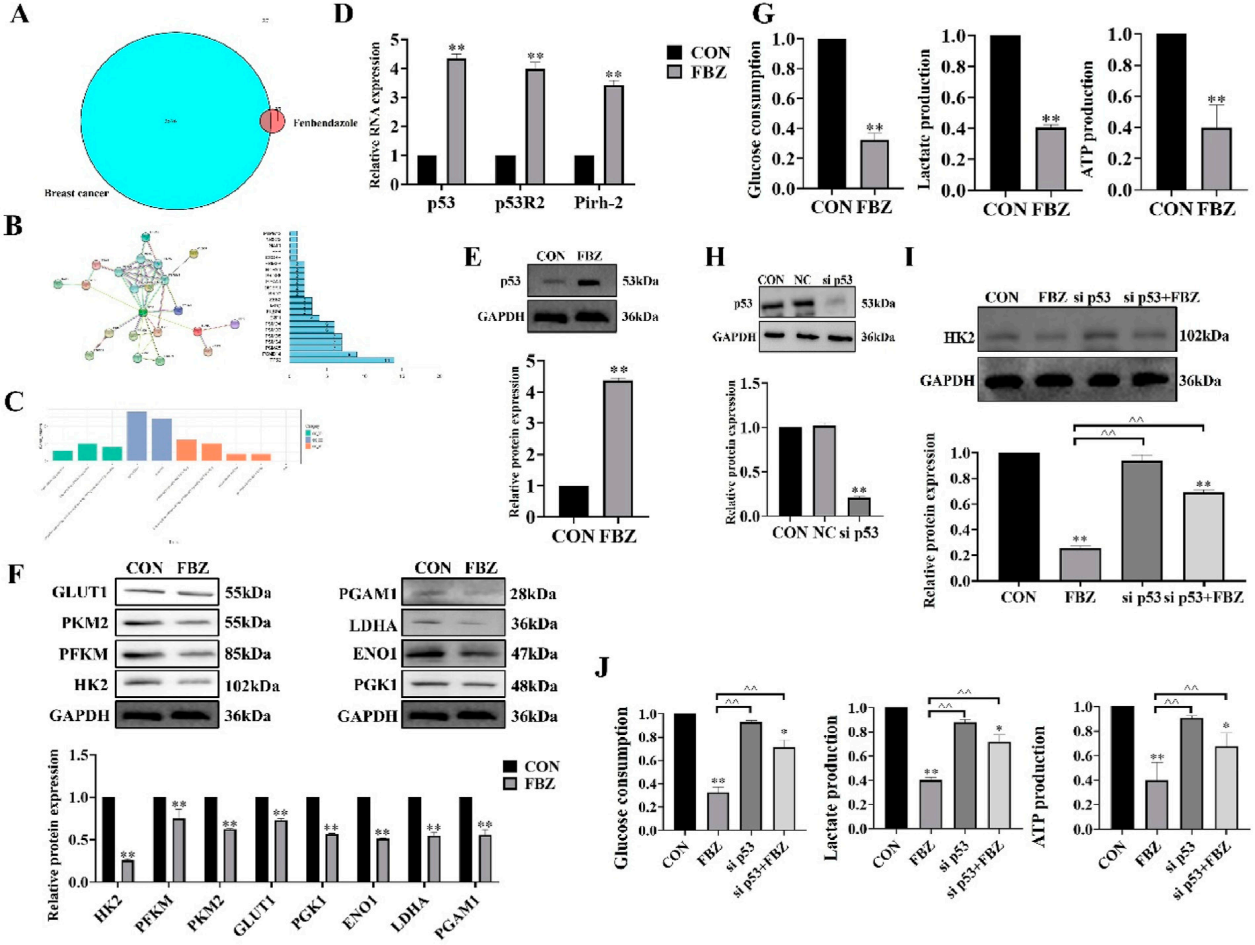
FBZ suppresses aerobic glycolysis in EMT6 cells via the p53-HK2 axis. **(A)** Network pharmacology of FBZ-breast cancer interactions. **(B)** PPI (Protein-Protein Interaction) network. (n = 3, FDR-corrected P < 0.05). **(C)** GO enrichment analysis of FBZ-downregulated proteins. **(D)** p53 pathway mRNA expression (qPCR, 2 μM FBZ, 24 h). (P < 0.05 vs. CON, n = 3, normalized to GAPDH). **(E)** p53 protein validation (WB, 2 μM FBZ, 24 h). (n = 3, normalized to GAPDH). **(F)** Glycolytic enzyme protein levels (WB, 2 μM FBZ, 24 h). (P < 0.05 vs. CON, n = 3, normalized to GAPDH). **(G)** Glycolytic function assays (2 μM FBZ, 24 h). (n = 6, mean ± SD). **(H)** p53 knockdown efficiency (siRNA). Groups: CON, NC (Negative Control), sip53. WB markers: p53 (53 kDa), GAPDH (36 kDa). (P < 0.01 vs. CON/NC, n = 3, normalized to GAPDH). **(I)** HK2 rescue experiment (siRNA + FBZ, 2 μM, 24 h). (n = 3, normalized to GAPDH). **(J)** Glycolytic parameters after p53 manipulation (2 μM FBZ, 24 h). (n = 6, mean ± SD).

To elucidate the mechanism of FBZ - induced pyroptosis, we first identified FBZ - interacting targets via network pharmacology and protein - protein interaction analyses (Figures 7A,B). GO enrichment analysis revealed FBZ - upregulated genes were enriched in the p53 signaling pathway (Figure 7C). Given the established link between p53 and tumor glycolysis, we hypothesized FBZ induces pyroptosis by modulating glycolysis via p53. qPCR assays showed upregulated transcript levels of p53 pathway - related genes (Figure 7D). WB analysis revealed FBZ significantly upregulated p53 protein in EMT6 cells (Figure 7E). Among eight key glycolytic enzymes, WB demonstrated FBZ downregulated their expression to varying degrees, with the most potent inhibition on HK2 (Figure 7F). FBZ also suppressed glucose consumption, lactate production, and ATP levels in EMT6 cells (Figure 7G). Notably, p53 knockdown partially reversed FBZ - mediated inhibition of HK2 expression, glucose consumption, lactate production, and ATP content (Figures 7H–J). These data collectively suggest FBZ induces pyroptosis by targeting the p53 - glycolysis axis, with HK2 as a critical downstream effector.

### 2.7 FBZ activated the BAX/caspase 3/GSDMF cascade by inhibiting HK-II

**FIGURE 8:**
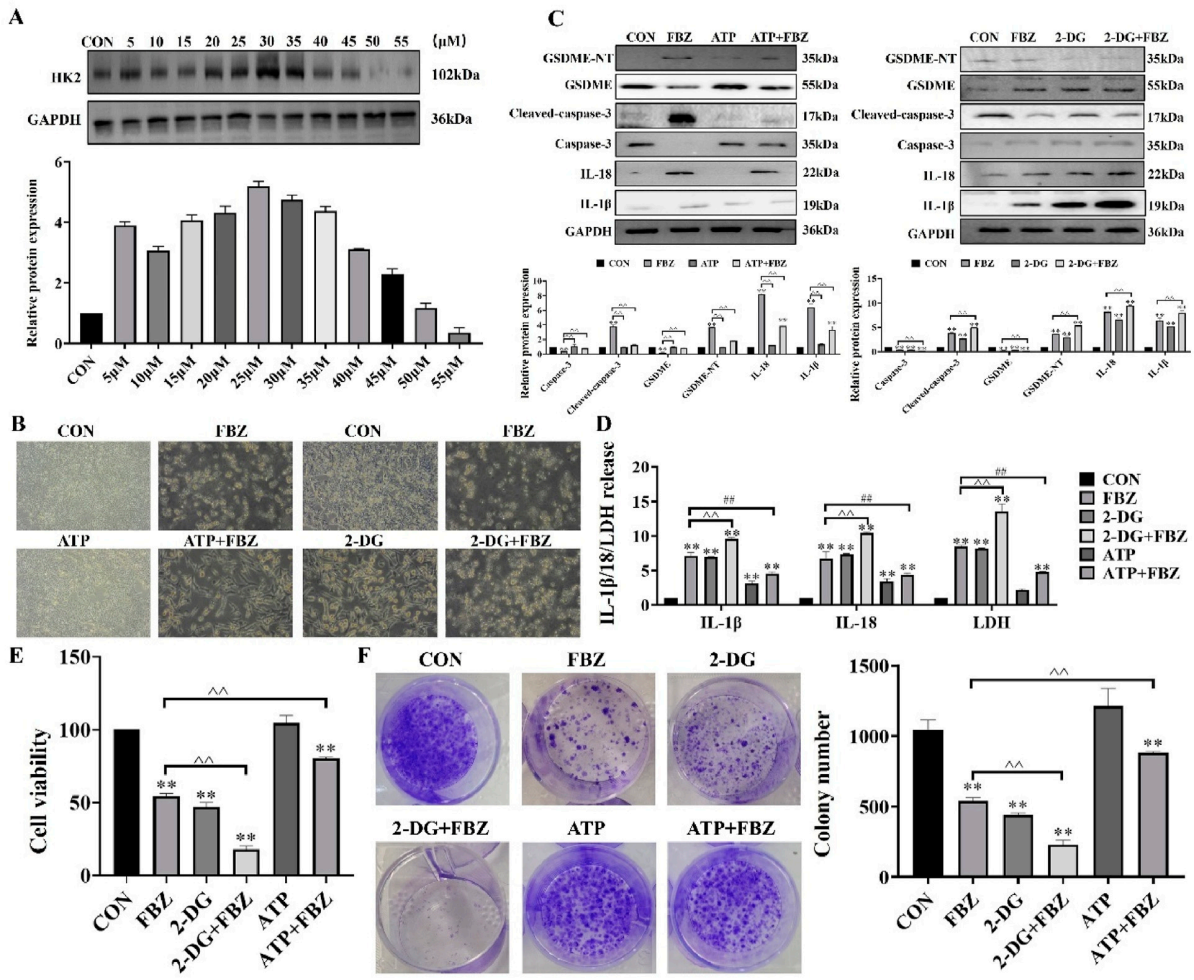
Relationship between HK2 and pyroptosis. **(A)** Detection of ATP use concentration (n = 3, mean ± SD). **(B)** Morphological observation. **(C)** Effect of ATP and 2-DG on caspase-3 and GSDME protein expression. **(D)** Detection of LDH, IL-18 and IL-1β release (n = 3, mean ± SD). **(E)** Detection of cell viability (n = 3, mean ± SD). **(F)** Detection of colony formation (n = 3, mean ± SD). 2 - DG (65 μM, 24 h pre - treatment) (Zhang et al., 2019) + FBZ (2 μM). ATP (30 μM, 24 h pre - treatment) (Figure 8A) + FBZ (2 μM) (n = 3, mean ± SD).

To investigate the role of HK2 in FBZ-induced pyroptosis, we modulated HK2 activity in EMT6 cells using 2-DG (HK2 inhibitor) and ATP (HK2 activator). The rationale for using 2-DG and ATP was to directly manipulate HK2 function and observe its impact on pyroptosis pathways, as 2-DG blocks HK2-mediated glycolysis while ATP supports HK2 activity. Morphological observations (Figure 8B) revealed that 2-DG enhanced FBZ-induced cell swelling and rupture, a typical pyroptotic feature, whereas ATP partially reversed these changes. Biochemical assays (Figures 8C,D) demonstrated that 2-DG + FBZ treatment significantly increased LDH release, IL-1β and IL-18 secretion, and the cleavage of GSDME-NT and cleaved-caspase-3 compared to FBZ alone. Conversely, ATP + FBZ treatment showed the opposite trend, with reduced pyroptotic marker levels. To further validate the functional consequences of HK2 modulation on cell fate, cell viability assays (Figure 8E) were performed. The data showed that 2-DG augmented FBZ-induced cell death, consistent with the pro-pyroptotic role of HK2 inhibition, while ATP partially rescued cell viability, suggesting that maintaining HK2 activity suppresses lytic cell death. Colony formation assays (Figure 8F) extended these findings: 2-DG + FBZ strongly suppressed colony formation, indicating that pyroptosis impairs the long-term proliferative capacity of cells, whereas ATP + FBZ partially restored clonogenic potential, linking HK2 activity to the preservation of viable, undamaged cells. Collectively, these data suggest that FBZ induces pyroptosis in EMT6 cells by inhibiting HK2, and modulating HK2 activity directly influences the pyroptotic response and long-term clonogenic potential.

## 3 Discussion

Complex mechanisms of drug resistance make the treatment of breast cancer a challenge (Will et al., 2023). Pyroptosis is a newly discovered programmed cell death that offers several targets for cancer treatment (Chen et al., 2022). Tumor glycolysis is a potential process for anti-tumor resistance (Aft et al., 2003). FBZ has been found to have good anti-tumor activity (Villar et al., 2007), but whether it can inhibit the growth of breast cancer through pyroptosis and glycolysis remains to be explored in detail. In the present study, we have demonstrated for the first time that FBZ inhibits the tumor glycolysis-induced caspase-3/GSDME pathway for pyroptosis in EMT6 cells.

In addition to its use as an antiparasitic agent, FBZ has also been found to have an anti-cancer effect. In non-small cell lung cancer, FBZ inhibited growth by increasing p53 activation, inhibiting the transporter protein GLUT1 and hexokinase, and reducing glucose uptake in cancer cells (Xu et al., 2022). In addition to inhibiting glycolysis, FBZ also induced apoptosis. In colorectal cancer (CRC) cells, FBZ induced apoptosis through mitochondrial damage and the caspase-3-PARP pathway (Jing et al., 2021). In wild-type CRC, FBZ activated apoptosis by increasing p53 expression. In 5-FU-resistant CRC, FBZ induced apoptosis without affecting p53 expression and could enhance p53-independent iron death to promote apoptosis (Park et al., 2022). In this study, we have shown that FBZ induces pyroptosis in mouse mammary xenografts and EMT6 cells. In addition, FBZ can impair microtubule polymerization and inhibit mTOR signaling to suppress ovarian cancer growth (Shin et al., 2023). In animals, FBZ has shown a high safety profile and low toxicity. A study on the safety profile of FBZ in cattle showed that FBZ was well tolerated even when administered at six times the prescribed dose and three times the recommended duration (Muser and Paul, 1984). In rodents, the lethal dose is more than 10 g/kg, which corresponds to 1,000 times the therapeutic amount (Muser and Paul, 1984). In this study, we confirmed the high safety of FBZ in mice by weighing the mice and determining the weights of the liver and kidneys, performing histopathologic sections of the liver and kidneys, and determining key biochemical indices of the liver and kidneys. In an *in vitro* model, we found that FBZ was practically non-toxic to HC11 cells. A major challenge in the use of FBZ is its low water solubility and bioavailability. Improving water solubility is crucial as it reduces the amount of drug needed to achieve the same therapeutic effect.

Pyroptosis has a profound effect on the invasion, proliferation and metastasis of tumor cells and is therefore of therapeutic importance in a variety of malignant diseases. According to several published studies, pyroptosis-associated modulators show a tumor suppressive effect in breast, liver, lung adenocarcinoma and bladder cancer (Hou et al., 2020; Derangère et al., 2014; Lin et al., 2021; Zhang et al., 2022). For example, DDP induced pyroptosis in triple negative breast cancer (TNBC) to inhibit tumor growth and metastasis (Yan et al., 2021). The natural product tretinoin induced pyroptosis in head and neck cell carcinomas to inhibit their growth (Cai et al., 2021). In our study, we found that FBZ induced pyroptosis in mouse breast xenografts and EMT6 cells through cell viability assay, tumor intuition and pyroptosis-related indices. GSDMs is an important protein that mediates pyroptosis, triggers cell death and promotes the release of inflammatory factors. It consists of six main components: Gasdermin A (GSDMA), Gasdermin B (GSDMB), Gasdermin C (GSDMC), Gasdermin D (GSDMD), GSDME and DFNB58. GSDMD is the most commonly used factor in tumor pyroptosis studies. In colon cancer, simvastatin induced pyroptosis via the caspase-1/GSDMD pathway (Xie et al., 2023). Carotenoid B triggered TLR4-induced pyroptosis via the GSDMD pathway in non-small cell lung cancer (Yuan et al., 2021). However, our data showed that FBZ could not significantly induce the activation of GSDMD in EMT6 cells and mouse breast xenografts. GSDME is a key protein in the switch between apoptosis and cellular pyroptosis and has been identified as a potential tumor suppressor gene. In melanocytomas, triggering GSDME-induced pyroptosis inhibited tumor growth and increased GSDME expression increased the sensitivity of melanocytomas to etoposide (Lage et al., 2001). The epigenetic silencing of GSDME was first discovered in primary gastric cancer. The introduction of GSDME into gastric cancer tumor cells inhibited their growth, suggesting that GSDME has oncogenic activity (Akino et al., 2007). Recent studies have shown that GSDME-expressing gastric cancer cells undergo pyroptosis when treated with chemotherapeutic agents such as 5-FU (Wang et al., 2018). However, in the absence of GSDME, 5-FU-induced apoptosis was converted to apoptosis, suggesting that GSDME converts chemotherapeutic agent-induced apoptosis into gastric cancer cell death (Jung et al., 2023). Our data showed that FBZ significantly activated caspase-3 and GSDME. When caspase-3 was inhibited or GSDME was turned off, FBZ-induced pyroptosis was attenuated. This suggests that FBZ triggers pyroptosis via the caspase-3/GSDME pathway. Our study as well as previous studies suggest that GSDME is an effective anti-tumour target.

Mutations in the p53 gene are important triggers for the development of more than 50% of human tumors. After years of research, it is now widely believed that wild-type p53 can inhibit tumor proliferation by blocking the cell cycle, promoting apoptosis, repairing damaged DNA, inhibiting tumor angiogenesis and other biological functions. This is in general agreement with previous findings that FBZ induced apoptosis in tumor cells by blocking them in the G2/M phase (Jung et al., 2023). Another study claimed that mebendazole, a benzimidazole analogue, inhibited angiogenesis in tumor tissue (Mukhopadhyay et al., 2002). It is possible that all these results are related to the fact that FBZ increases the expression of the wild-type p53 gene. EMT6 cells’ whose phenotype of p53 was wild-type were used in this experiment. Our results suggested that FBZ inhibit glycolysis-induced pyroptosis by regulating p53 gene.

The high demand for glucose is a main feature of tumor cells, which is also known as the Warburg effect (Zhu et al., 2022). Therefore, regulation of tumor development from this aspect can have a good inhibitory effect. For example, targeting ACYP1-mediated glycolysis can reverse lenvatinib resistance and limit the progression of hepatocellular carcinoma (Wang et al., 2023). Aerobic glycolysis controls myeloid-derived suppressor cells and tumor immunity in triple-negative breast cancer (Li et al., 2018). HK2 is a crucial enzyme in the glycolysis process and plays a significant role in this process (Padilla and Lee, 2021). Several drugs have been discovered to modulate the glycolysis process in tumor cells by targeting HK2. For instance, Xanthohumol has been shown to inhibit colorectal cancer cells by downregulating HK2-mediated glycolysis (Liu et al., 2019). Similarly, Tanshinone IIA has been found to reduce HK2-mediated aerobic glycolysis in oral squamous cell carcinoma (Li et al., 2020). In our study, we observed that FBZ regulated glycolysis in EMT6 cells through HK2 and induced pyroptosis in tumor cells. These findings, along with previous studies, highlight the importance of HK2 as a target for drug-induced tumor cell death and suggest its potential as a new avenue for clinical targeted therapy.

In summary, our study has demonstrated that FBZ has potential antitumour activity and can exert potent antitumour effects in terms of both tumor metabolism and tumor cell death.

## 4 Materials and methods

### 4.1 Cell culture

The mouse breast cancer cell line (EMT6) and the mouse mammary epithelial cell (HC11) were purchased from Wuhan Procell Life Science and Technology Co., Ltd (Wuhan, China). Both cells were subcultured in 1640 medium (Pricella, Wuhan, China), supplemented with 10% FBS (Pricella, Wuhan, China) and 0.1% penicillin–streptomycin solution (Pricella, Wuhan, China), and cultured in a cell incubator at 37°C under 5% CO_2_-saturated humidity. When the cell confluence reached about 70%–80%, the cells were digested with trypsin for subculture.

### 4.2 Animals

Balb/c mice (female, 6–8 weeks old, weighing 19 ± 1 g) were purchased from Liaoning Changsheng Co. The mice were housed in a mouse room at a temperature of 23°C ± 1°C. The bedding was changed regularly once a day, and the feeding cycle was set at 12 h intervals. During the feeding period, sufficient food was supplied and free access to water was provided.

### 4.3 Preparation solutions

The FBZ, adenosine triphosphate (ATP), and dimethyl sulfoxide (DMSO) used in the experiment were purchased from Solarbio (Beijing, China). Cisplatin (DDP) used in the experiment was purchased from Shanghai Yuanye Biotechnology Co., Ltd (Shanghai, China). Z-DEVD-FMK, ATP, and 2-DGwere purchased from GLPBIO (Montclair, CA, United States). FBZ, DDP, 2-DG, ATP, and Z-DEVE-FMK were dissolved in DMSO and then diluted with RPMI 1640 medium. The final concentration of DMSO in the working solution did not exceed 0.1% (Deng et al., 2014).

### 4.4 Cell counting Kit-8 (CCK8) assays and morphological observations

We seeded the cells in 96-well plates. At 80% confluence, the cells were treated with 0 μM, 0.5 µM, 2 μM, 8 µM FBZ; 10 µM Z-DEVD-FMK; 65 µM 2-DG; and 30 µM ATP. After the 22-h treatment, the cells were continuously cultured with 10 µL CCK8 in each well at 37°C for 2 h. After the 24-h treatment, the cells were photographed under the microscope.

### 4.5 Colony formation assay

We seeded the cells into 6-well plates. When the cell confluence reached 80%, the cells were treated with various drugs. Following the treatment, the cells were fixed with paraformaldehyde for 15 min. Subsequently, 0.1% crystal violet solution was added to each well, and the staining was carried out for 30 min. Finally, images of the stained cells were captured.

### 4.6 Real-time fluorescence quantitative PCR (qPCR)

Total cellular RNA was extracted using Trizol reagent (Invitrogen/Thermo Fisher Scientific, Waltham, MA, United States). Following reverse transcription of the extracted RNA, quantitative real-time PCR (qPCR) was performed using SYBR Premix Ex Taq (#RR420A; Takara, Otsu, Japan) on a 7,500 Fast Real-Time PCR System (Applied Biosystems, Foster City, CA, United States) (Table 1).

**TABLE 1:** qPCR primer sequences.

Gene name	Gene sequence
Caspase-1	Forward Primer: AATACAACCACTCGTACACGTC Reverse Primer: AGCTCCAACGCTCGGAGAAA
Caspase-3	Forward Primer: ATGGAGAACAACACAACCTCAGT Reverse Primer: TTGCTCCCATGTATGGTCTTTAC
Caspase-8	Forward Primer: TGCTTGGACTACATCCCACAC Reverse Primer: TGCAGTCTAGGAAGTTGACCA
GSDMA	Forward Primer: ACGCGGGGAGAACCTCTAT Reverse Primer: GCCGTTGACCATCAGTTGTCT
GSDMC	Forward Primer: ATGTCCTACACATTTGACTGGC Reverse Primer: TTAAACAGGCAGAATTTGGTTGC
GSDMD	Forward Primer: TTCAGGCCCTACTGCCTTCT Reverse Primer: GTTGACACATGAATAACGGGGTT
GSDME	Forward Primer: TCACCACGGACACCAATGTAG Reverse Primer: CCACCATGTCTCCCTTGGAG
IL - 1β	Forward Primer: TTCAGGCAGGCAGTATCACTC Reverse Primer: GAAGGTCCACGCGATAGACAC
IL - 18	Forward Primer: GGACTCTTGCGTCAACTTCAAGG Reverse Primer: CAGGCTGTCTTTTGTCAACGA
p53	Forward Primer: GCGTAAACGCTTCGAGATGTT Reverse Primer: TTTTTATGGCGGGAAGTAGACTG
p53R2	Forward Primer: CCTTGCGATGGATAGCAGATAGA Reverse Primer: GCCAGAATATAGCAGCAAAAGATC
pirh-2	Forward Primer: GGCTGTGTCACGATACCAATG Reverse Primer: GCTACAGTCTTCACAAGTCTGCT
GAPDH	Forward Primer: TGGCCTTCCGTGTTCCTAC Reverse Primer: GAGTTGCTGTTGAAGTCGCA

### 4.7 Western blot (WB)

After treatment with various drugs, cells were harvested and lysed in RIPA buffer (Yamei, Shanghai, China) supplemented with protease and phosphatase inhibitors (Yamei, Shanghai, China). The extracted protein samples were mixed with protein loading buffer and boiled in a metal bath at 100°C for 12 min. The samples were separated by SDS-PAGE and transferred to a PVDF membrane. After incubation with primary and secondary antibodies (Table 2), images were captured using an iBright imaging system (Thermo Fisher Scientific, Waltham, MA, United States).

**TABLE 2:** Antibody information.

Antibody name	Manufacturer, catalog number, dilution ratio
Caspase-1	ABclonal, A0964,1:1000
Caspase-3	ABclonal, A25309,1:1000
Caspase-8	ABclonal, A0251,1:1000
GSDMA	ABclonal, A22624,1:1000
GSDMC	ABclonal, A14550,1:1000
GSDME	ABclonal, A25293,1:1000
IL - 1β	ABclonal, A16288,1:1000
IL - 18	ABclonal, A23076,1:1000
BAX	ABclonal, A12009,1:1000
p53	ABclonal, A19836,1:1000
HK2	ABclonal, A20829,1:1000
PFKM	ABclonal, A3671,1:1000
PKM2	ABclonal, A0268,1:1000
GLUT1	ABclonal, A6982,1:1000
PGK1	ABclonal, A12686,1:1000
ENO1	ABclonal, A16841,1:1000
LDHA	ABclonal, A21893,1:1000
PGAM1	ABclonal, A4170,1:1000
GAPDH	ABclonal, AC002,1:1000
Goat Anti-Mouse IgG (H + L)	UpingBio, YP848536,1:5000
Goat Anti-Rabbit IgG (H + L)	ABclonal, RM70003,1:5000

### 4.8 Transfection

Cells were seeded in 6-well plates at a density designed to achieve 70% confluence prior to transfection. Transfection was performed using Lipofectamine 3000 (Invitrogen, Carlsbad, CA, United States) to deliver siRNAs (Sangon Biotech, Shanghai, China) (Table 3) targeting GSDME, BAX, p53, or a non-targeting negative control (NC) siRNA. The transfection protocol strictly followed the manufacturer’s guidelines: siRNAs were pre-diluted in Opti-MEM medium and mixed with Lipofectamine 3000 reagent for 15 min at room temperature to form transfection complexes, which were then added to the wells. Cells were incubated for 48 h post-transfection to allow efficient gene silencing before subsequent treatments.

**TABLE 3:** siRNA Sequences.

Name	Sequence
GSDME-1	Sense Strand: 5′-GCAGCUUGUGGUACUGGAATT-3′ Antisense Strand: 5′-UUCCAGUACCACAAGCUGCTT-3′ NC: 5′-UUCUCCGAACGUGUCACGUTT-3′
GSDME-2	Sense Strand: 5′-GGAAGACACAGCUGAUGAATT-3′ Antisense Strand: 5′-UUCUCCAGUACCACAAGCTT-3′ NC: 5′-UUCUCCGAACGUGUCACGUTT-3′
BAX-1	Sense Strand:5′-GAGCUGACAUCCAGGAUGATT-3′ Antisense Strand: 5′-UCATCCTGGATGTCAGCTCTT-3′ NC:5′-UUCUCCGAACGUGUCACGUTT-3′
BAX-2	Sense Strand:5′-CCACAAGAAGCUGAGCGAGTT-3′ Antisense Strand: 5′-CUCGCTCAGCTTCTTGTGGTT-3′ NC:5′-UUCUCCGAACGUGUCACGUTT-3′
p53-1	Sense Strand: 5′-GCACAAACAGACGCCUAUGTT-3′ Antisense Strand: 5′-CAUAGGCGUCUGUUUGUGCTT-3′ NC: 5′-UUCUCCGAACGUGUCACGUTT-3′
p53-2	Sense Strand: 5′-GGAGAAGACCUCCAAGAAATT-3′ Antisense Strand: 5′-UUUCUUGGAGGUCUUCUCCTT-3′ NC: 5′-UUCUCCGAACGUGUCACGUTT-3′

### 4.9 Glucose, lactate, ATP, lactate dehydrogenase (LDH) IL-1β, IL-18 detection

Cells were seeded in six-well plates until they were 90% confluent. Test after dosing according to the instructions (Elabscience, Wuhan, China).

### 4.10 Blood tests

Mouse blood was collected and placed in a centrifuge tube containing anticoagulant. Automatic detection according to the blood cytometry programme.

### 4.11 Liver and kidney biochemical indicator tests

Blood was collected from the eye sockets of mice in clean, enzyme-free centrifuge tubes, and serum levels of AST, ALT, BUN and CREA were determined using an automated biochemical analyzer (Beckman CX7, Chaska, America).

### 4.12 Hematoxylin-eosin staining (HE)

Liver and kidney tissues were collected and fixed in 10% formaldehyde solution before paraffin sectioning and HE staining (Boster, Wuhan, China).

### 4.13 Cyberpharmacology

The GeneCards, PharmMapper, NCBI, and DAVID 6.8 databases were utilized to identify target genes associated with breast tumors and modulated by FBZ. A Venn diagram was created to visualize the results of the GO enrichment analysis.

### 4.14 Statistical analyses

Statistical analyses were performed using IBM SPSS Statistics (version 22) and GraphPad Prism (version 8) software. The data were derived from three independent experiments. The descriptive statistics function was used to calculate the mean and standard deviation (SD), and the results were visualized as error bars in the figures. A two-tailed t-test was used to assess significant differences between the tested samples and the control group, with p-values calculated accordingly. A p-value <0.05 was considered statistically significant. Data are presented as means ± SD, where * denotes p < 0.05, ** denotes p < 0.01, ˆ denotes p < 0.05, and ˆˆ denotes p < 0.01.

## 5 Conclusion

FBZ induces pyroptosis and inhibits aerobic glycolysis in mouse EMT6 cells by targeting HK2, a key regulator linking metabolic reprogramming to pyroptotic cell death.

## Data Availability

The original contributions presented in the study are included in the article/Supplementary Material, further inquiries can be directed to the corresponding authors.

## Glossary

2-DG: 2-Deoxy-D-Glucose
ALT: Alanine Aminotransferase
AST: Aspartate Aminotransferase
BAX: Bcl-2-Associated X Protein
BUN: Blood Urea Nitrogen
CCK-8: Cell Counting Kit-8
CREA: Creatinine
DAMPs: Damage-Associated Molecular Patterns
DFNA5: Deafness, Autosomal Dominant 5
DDP: Cisplatin
DMSO: Dimethyl sulfoxide
EMT6: Mouse Mammary Carcinoma Cell Line
FBZ: Fenbendazole
FBS: Fetal Bovine Serum
GAPDH: Glyceraldehyde 3-Phosphate Dehydrogenase
GO: Gene Ontology
GSDM: Gasdermin
HK2: Hexokinase 2
HC11: Mouse Mammary Epithelial Cell Line
HE: Hematoxylin-Eosin (staining)
IL-1β: Interleukin-1 beta
IL-18: Interleukin-18
LDH: Lactate Dehydrogenase
LDHA: Lactate Dehydrogenase A
NC: Negative Control (siRNA)
PAMPs: Pathogen-Associated Molecular Patterns
PFKM: Phosphofructokinase, Muscle
PGAM1: Phosphoglycerate Mutase 1
PGK1: Phosphoglycerate Kinase 1
PKM2: Pyruvate Kinase M2
PPI: Protein-Protein Interaction
qPCR: Quantitative Real-Time Polymerase Chain Reaction
siRNA: Small Interfering Ribonucleic Acid
WB: Western Blot
Z-DEVD-FMK: Caspase-3 Specific Inhibitor
