# Supplementary figures and images for: Fenbendazole induces pyroptosis in breast cancer cells through HK2/caspase-3/GSDME signaling pathway
Source: Front Pharmacol. 2025 Jul 18;16:1596694. doi: 10.3389/fphar.2025.1596694 (PMC12314287; doi:10.3389/fphar.2025.1596694)

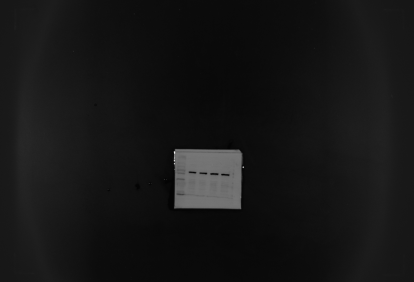

Supplement: Supplementary file 1 [file DataSheet3.zip › Caspase-3/caspase-3-1(siBAX).png]

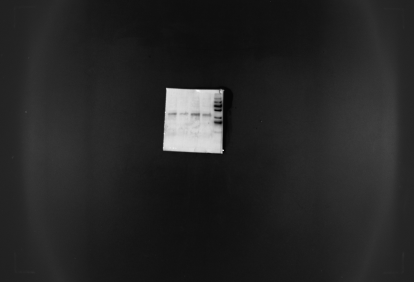

Supplement: Supplementary file 1 [file DataSheet3.zip › Caspase-3/caspase-3-2(siBAX).png]

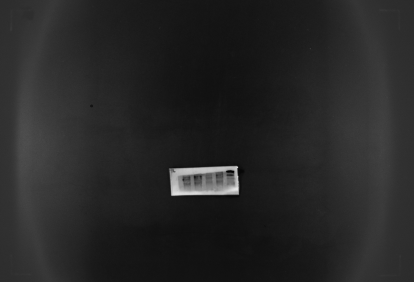

Supplement: Supplementary file 1 [file DataSheet3.zip › Caspase-3/caspase-3(siBAX).png]

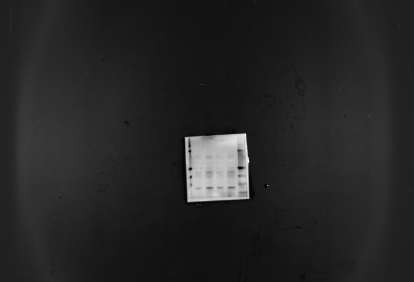

Supplement: Supplementary file 1 [file DataSheet3.zip › Caspase-3/cleaved-caspase-3(siBAX).png]

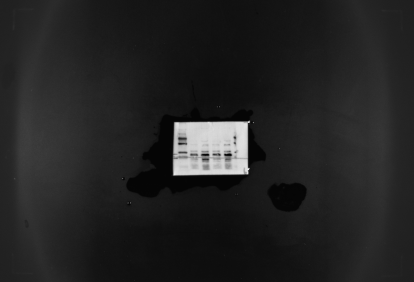

Supplement: Supplementary file 1 [file DataSheet3.zip › Caspase-3/cleaved-caspase-3-1(siBAX).png]

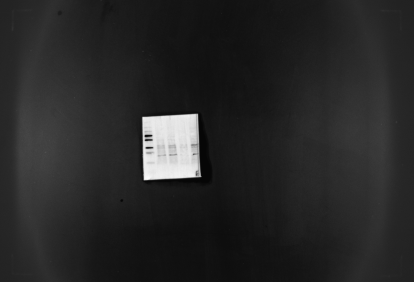

Supplement: Supplementary file 1 [file DataSheet3.zip › Caspase-3/cleaved-caspase-3-2(siBAX).png]

GSDMA (CON/FBZ）


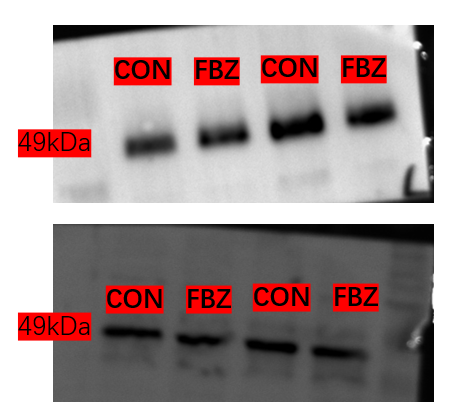


si GSDME


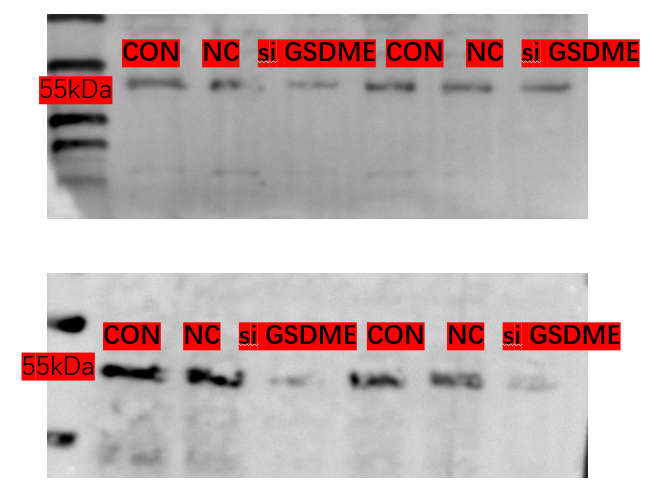


si BAX


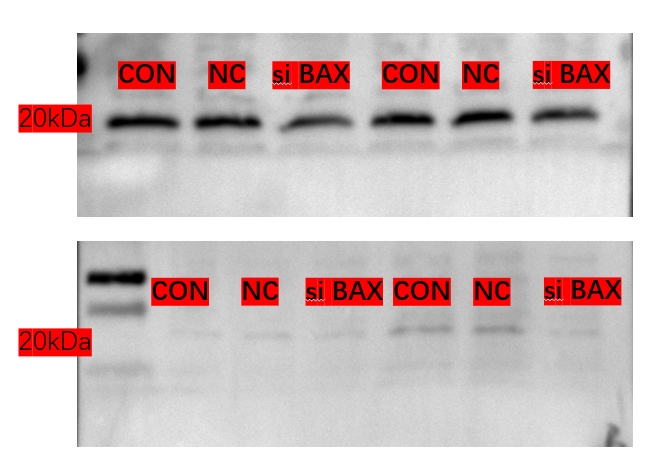


Caspas-3


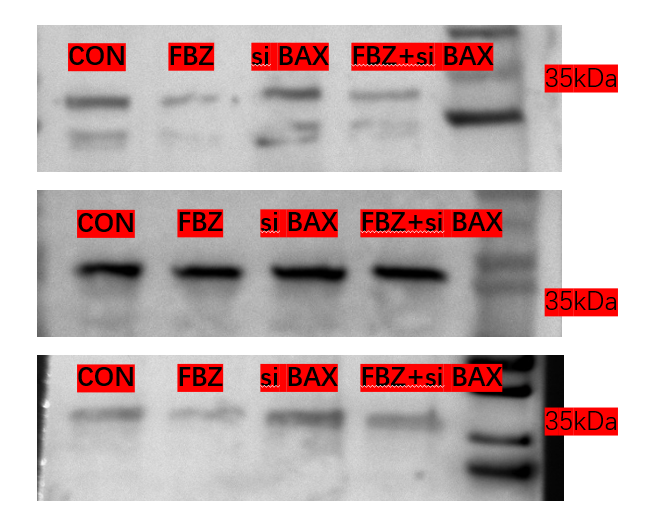


Cleaved-caspase-3


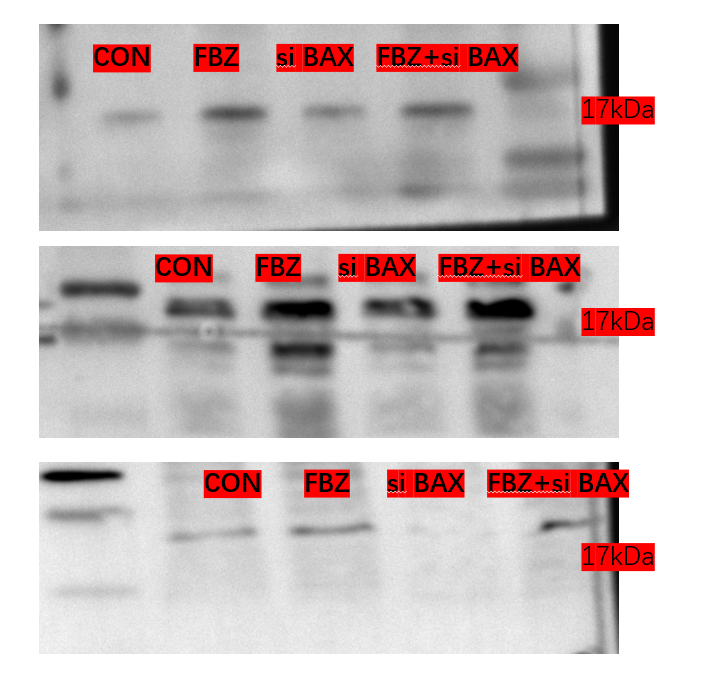


GSDME


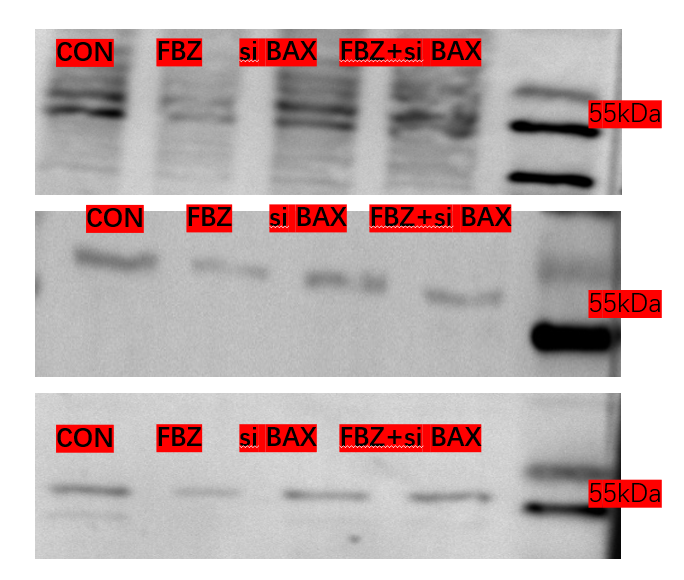


GSDME-NT


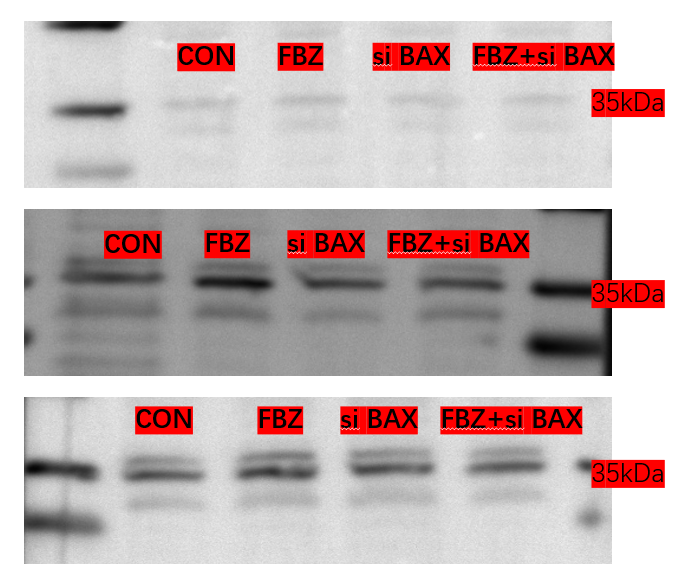


si p53


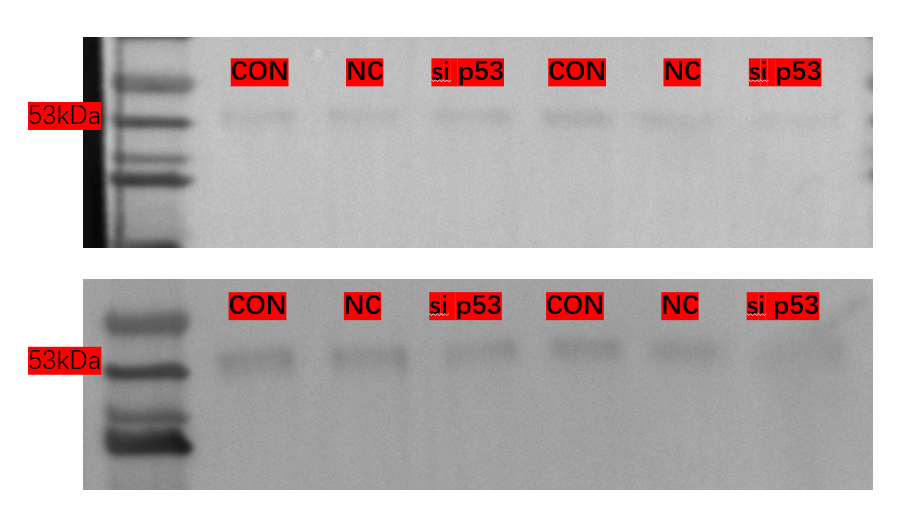


HK2


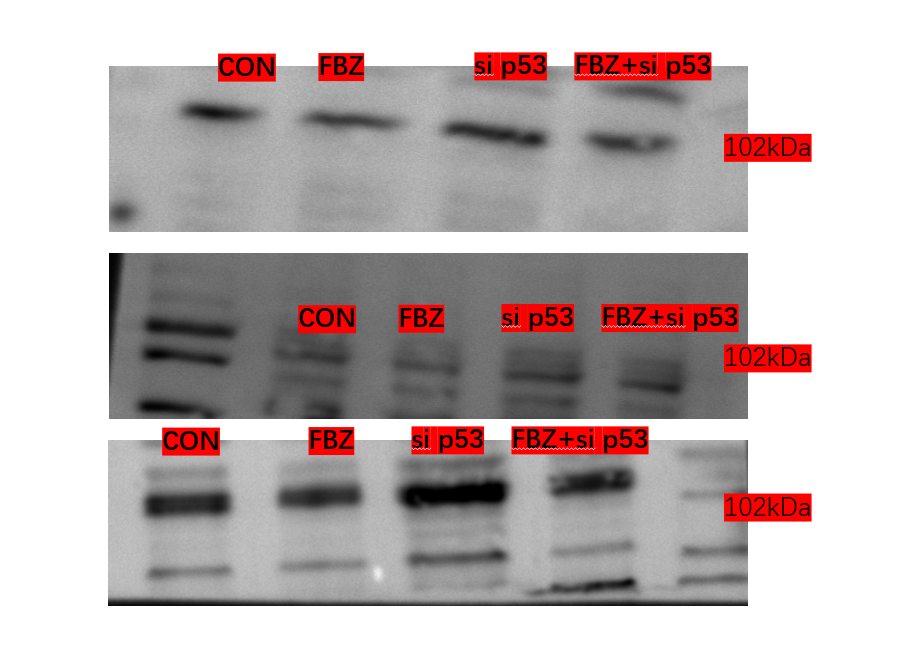

Supplement: Supplementary file 2 [file Table1.docx]

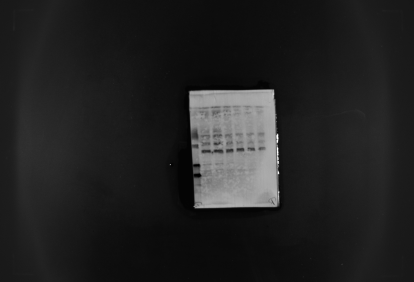

Supplement: Supplementary file 3 [file DataSheet4.zip › sip53/GAPDH-1.png]

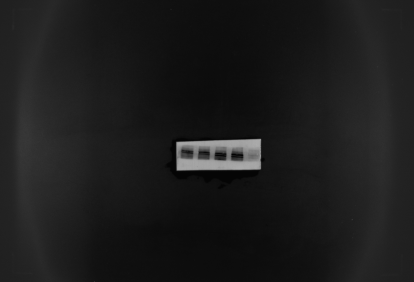

Supplement: Supplementary file 3 [file DataSheet4.zip › sip53/HK2/GAPDH.png]

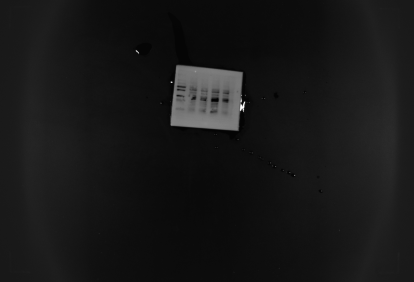

Supplement: Supplementary file 3 [file DataSheet4.zip › sip53/HK2/HK2-1.png]

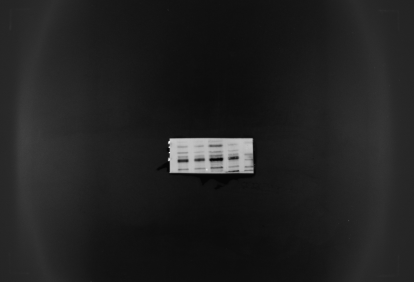

Supplement: Supplementary file 3 [file DataSheet4.zip › sip53/HK2/HK2-2.png]

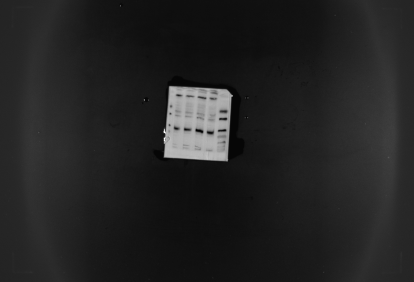

Supplement: Supplementary file 3 [file DataSheet4.zip › sip53/HK2/HK2.png]

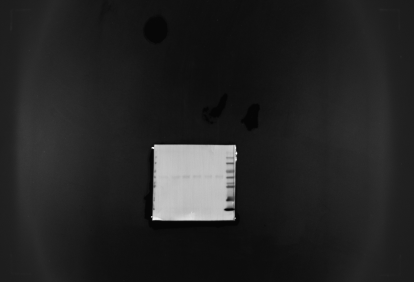

Supplement: Supplementary file 3 [file DataSheet4.zip › sip53/p53-1.png]

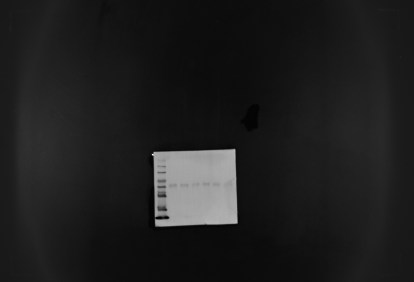

Supplement: Supplementary file 3 [file DataSheet4.zip › sip53/p53.png]

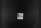

Supplement: Supplementary file 4 [file DataSheet1.zip › GSDMA/GAPDH(GSDMA).png]

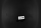

Supplement: Supplementary file 4 [file DataSheet1.zip › GSDMA/GSDMA-1.png]

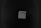

Supplement: Supplementary file 4 [file DataSheet1.zip › GSDMA/GSDMA.png]

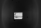

Supplement: Supplementary file 4 [file DataSheet1.zip › si GSDME/GAPDH(si GSDME).png]

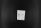

Supplement: Supplementary file 4 [file DataSheet1.zip › si GSDME/GSDME-1.png]

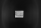

Supplement: Supplementary file 4 [file DataSheet1.zip › si GSDME/GSDME.png]

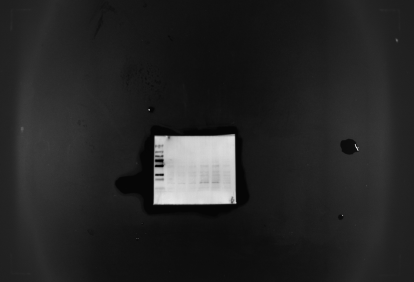

Supplement: Supplementary file 6 [file DataSheet2.zip › siBAX/BAX-1.png]

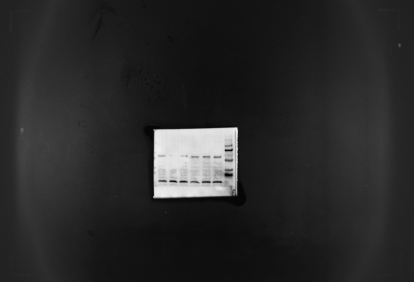

Supplement: Supplementary file 6 [file DataSheet2.zip › siBAX/BAX.png]

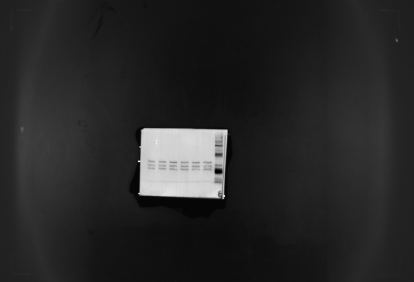

Supplement: Supplementary file 6 [file DataSheet2.zip › siBAX/GAPDH(si BAX).png]

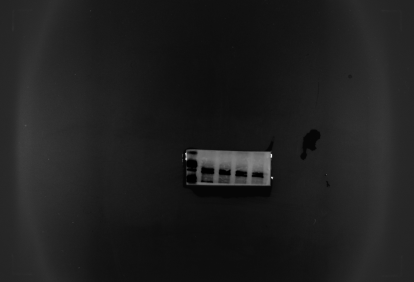

Supplement: Supplementary file 7 [file DataSheet5.zip › GSDME/GAPDH(siBAX).png]

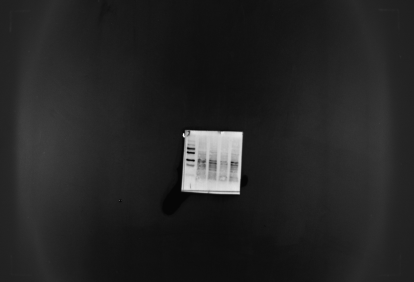

Supplement: Supplementary file 7 [file DataSheet5.zip › GSDME/GSDME(siBAX).png]

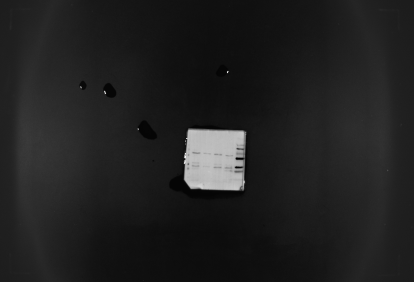

Supplement: Supplementary file 7 [file DataSheet5.zip › GSDME/GSDME-1(siBAX).png]

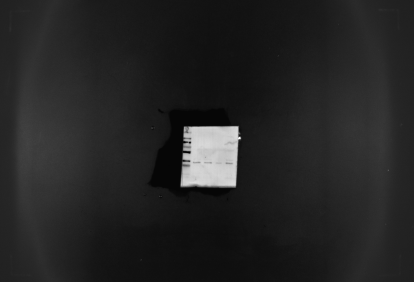

Supplement: Supplementary file 7 [file DataSheet5.zip › GSDME/GSDME-2(siBAX).png]

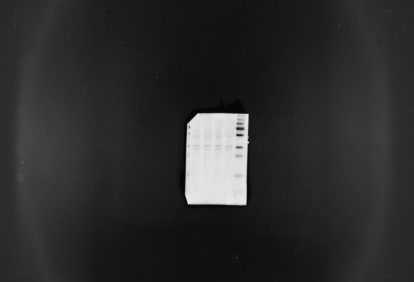

Supplement: Supplementary file 7 [file DataSheet5.zip › GSDME/GSDME-NT(siBAX).png]

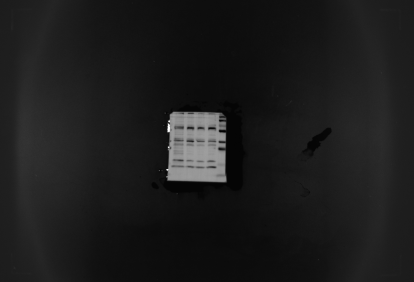

Supplement: Supplementary file 7 [file DataSheet5.zip › GSDME/GSDME-NT-1(siBAX).png]

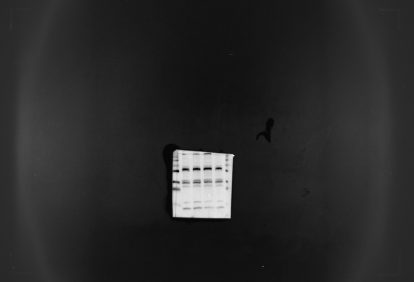

Supplement: Supplementary file 7 [file DataSheet5.zip › GSDME/GSDME-NT-2(siBAX).png]

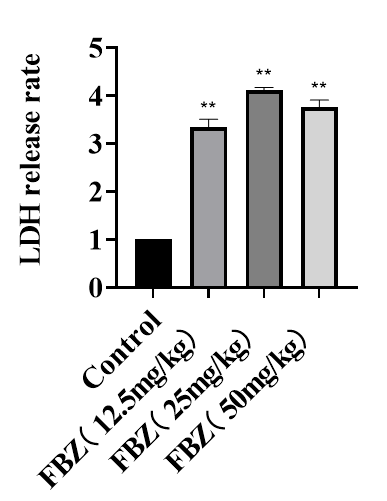

Supplement: Supplementary file 8 [file DataSheet7.zip › S1.png]
